# The origin of the exceptionally low activation energy of oxygen vacancy in tantalum pentoxide based resistive memory

**DOI:** 10.1038/s41598-019-53498-3

**Published:** 2019-11-19

**Authors:** Ji-Hyun Hur

**Affiliations:** 1 Department of Electrical Engineering, Sejong University, 209, Neungdong-ro, Gwangjin-gu, Seoul, 05006 Republic of Korea; 2 Hur Advanced Research, 96, Dongtanbanseok-ro, Hwaseong-si, Gyeonggi-do, 18456 Republic of Korea

**Keywords:** Electronic devices, Electronic devices, Electronic properties and materials, Electronic properties and materials

## Abstract

It is well known that collective migrations of oxygen vacancies in oxide is the key principle of resistance change in oxide-based resistive memory (OxRAM). The practical usefulness of OxRAM mainly arises from the fact that these oxygen vacancy migrations take place at relatively low operating voltages. The activation energy of oxygen vacancy migration, which can be inferred from the operational voltage of an OxRAM, is much smaller compared to the experimentally measured activation energy of oxygen, and the underlying mechanism of the discrepancy has not been highlighted yet. We ask this fundamental question in this paper for tantalum oxide which is one of the most commonly employed oxides in OxRAMs and try the theoretical answer based on the first-principles calculations. From the results, it is proven that the exceptionally large mobility of oxygen vacancy expected by the switching model can be well explained by the exceptionally low activation barrier of positively charged oxygen vacancy within the two-dimensional substructure.

## Introduction

In recent decades, oxide-based resistance switching nanodevice, often called OxRAM (oxide-based resistive random access memory) has been the subject of numerous semiconductor memory manufacturers and academies due to its potential advantages in good scalability, high endurance of switching, and fast switching speed. OxRAMs usually have bi-layer oxide structures, in which a few nanometer thick, nearly stoichiometric oxide layer (resistance switching layer) with higher resistivity and a more thick, metal-rich layer with lower resistivity (base layer) are sandwiched by two electrodes^1–11^. OxRAMs have demonstrated endurance of up to 10^11^, which is much larger than NAND flash and comparable to DRAM and also mark faster switching speed, usually in the range of 100 ns, or even under nanosecond^1^. High switching speed of OxRAMs, combined with lower operation voltage than NAND flash also allows for low program/erase power consumption which is especially suitable for low-power applications.

Among those oxides typically deployed in OxRAMs as resistance changing materials, tantalum pentoxide (Ta_2_O_5_) is one of the most popular one because Ta_2_O_5_-based OxRAMs have demonstrated the highest level of performance among all kind of OxRAMs^2–9^. Furthermore, the device performances reported in the lots of studies are quite uniform^4,5,7–10^, compared to the other kind of OxRAMs. And this relatively good reproducibility of Ta_2_O_5_-based OxRAMs is one of the motivations for this study to identify the huge discrepancy in the material property of Ta_2_O_5_ between the estimation from the OxRAM operations and what has been known from the typical measurements.

Given the fact that resistance switching in an OxRAM is caused by migrations of O atoms through vacancies^2–6^, the migration characteristics of O vacancy within the OxRAM is one of the most important material properties of the OxRAM determining the key features of it. However, despite its importance, activation energy of O vacancy itself in OxRAMs has been no serious study topics at all. Although recently there was a theoretical report about O vacancy activation barrier in Ta_2_O_5_^12^, it only deals with a sufficiently thick Ta_2_O_5_ film that inevitably involves inter-layer migrations which is not the case for OxRAM consisting of a nanometer-thin Ta_2_O_5_ film. In this manner, the estimated, unusually small activation energy barrier of O vacancy in Ta_2_O_5_-based OxRAMs has been just accepted and neglected to understand the underlying origin. As a matter of fact, there is a huge difference between the estimated activation energy of O vacancy from the switching characteristics of Ta_2_O_5_-based OxRAMs^3,5,6^ and the values obtained by the typical measurement methods^11,13,14^. Obviously, this small activation barrier of O vacancy expected in Ta_2_O_5_-based OxRAMs plays a central role in allowing a Ta_2_O_5_-based OxRAM to operate at low voltages and thus at low power consumption mode. Nonetheless, there still is no clue as to why the energy barrier is significantly lower than in normal situations.

In this paper, we theoretically investigate the activation energy of O vacancies in Ta_2_O_5_ particularly for the orthorhombic λ phase which is believed to be the representative crystal structure in energy stability. Although recently there was a report about O vacancy activation barrier in the orthorhombic λ phase Ta_2_O_5_^12^, it deals with a bulk Ta_2_O_5_ that inevitably involves inter-layer O vacancy migrations which is not always the case for the resistance changing volume in OxRAMs. By means of analyzing all the most plausible cases, we find the decisive migration pathways for different circumstances and the activation energies of those that mainly determine the overall O vacancy diffusion characteristics. It is revealed that the situation can be greatly changed depending on the effective crystallinity of the film affected by the fabrication process and the size of the region of interest. The calculation results are verified by comparing with the experimental measurements which demonstrate the good agreements.

## Results

### Material model and computational details

It has been well known that Ta_2_O_5_ is stable at orthorhombic phase up to about 1350 °C and above that, tetragonal phase is more stable^15,16^. The atomic crystal structure of Ta_2_O_5_ had long been controversial, but recently the controversy has come to near end as the most energy stable orthorhombic λ phase model structure has been proposed^17^. The orthorhombic λ phase Ta_2_O_5_ (space group *Pbam*) consists of two formula units per unit cell most closely packed among the proposed models. The total energy per formula unit is lower than 11-formular unit model which was the most energy stable Ta_2_O_5_ structure by then. In this structure, there are three different types of O sites which are 2-fold coordinated inter-layer sites between the two-dimensional (2D) Ta_2_O_3_ layers, 2-fold intra-layer sites within the layers and 3-fold intra-layer sites within the layers, whereas Ta sites are all 6-fold coordinated.

**Figure 1:**
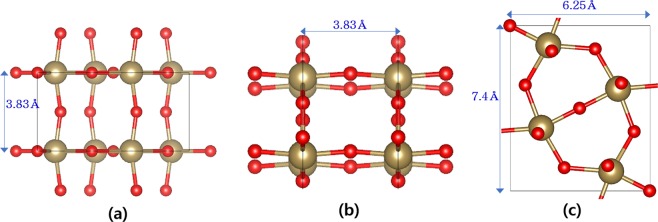
Atomic structure of the orthorhombic λ phase Ta_2_O_5_ with the space group symmetry of *Pbam* viewed along (**a**) x-axis, (**b**) y-axis, and (**c**) z-axis.

Figure 1 shows the crystal structure of the orthorhombic λ phase Ta_2_O_5_ from the three different view angles. The GGA-optimized lattice parameters of the cell structure are a = 6.25 Å, b = 7.4 Å, and c = 3.83 Å which coincide with the previous works^17–19^. To make supercells bearing one O vacancy, the intra-layer O vacancies are formed by removing one O atom in 2D layer of the Ta_2_O_3_ and inter-layer O vacancies are formed by removal of one of inter-layer O atoms bonding with Ta atoms. Therefore, basically there are three types of O vacancy migration pathways: (1) migration between two adjacent intra-layer O vacancy sites, (2) between neighboring two inter-layer O vacancy sites, and (3) between an inter-layer and an intra-layer O sites.

**Figure 2:**
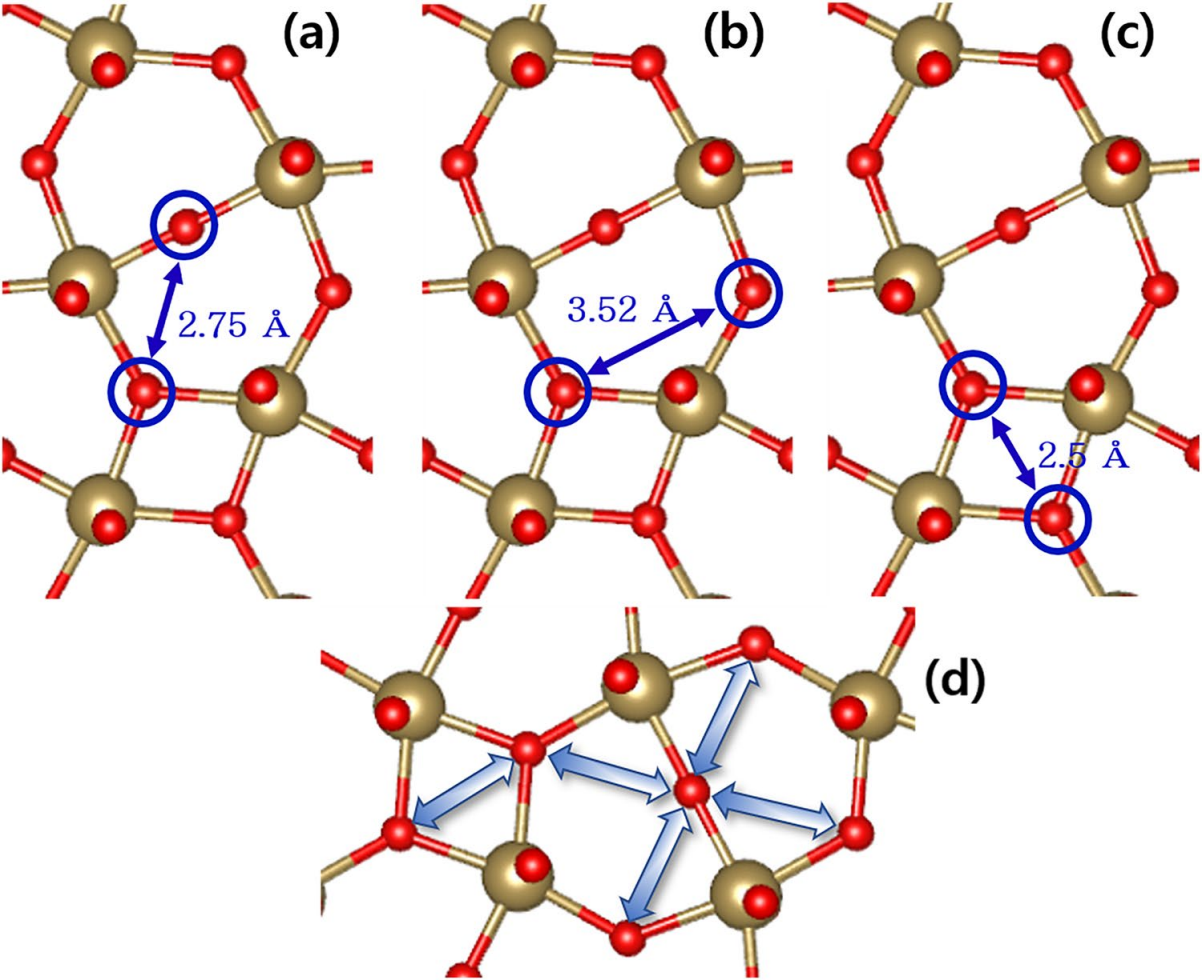
The possible O pairs participating vacancy migrations within the Ta_2_O_3_ layer. (**a**) Between 2-fold and 3-fold (A-path), (**b**) between the second nearest 3-fold (B-path), and (**c**) between the first nearest 3-fold (C-path) sites. (**d**) is an example of the self-connected pathway combining A- and B-paths.

**Figure 3:**
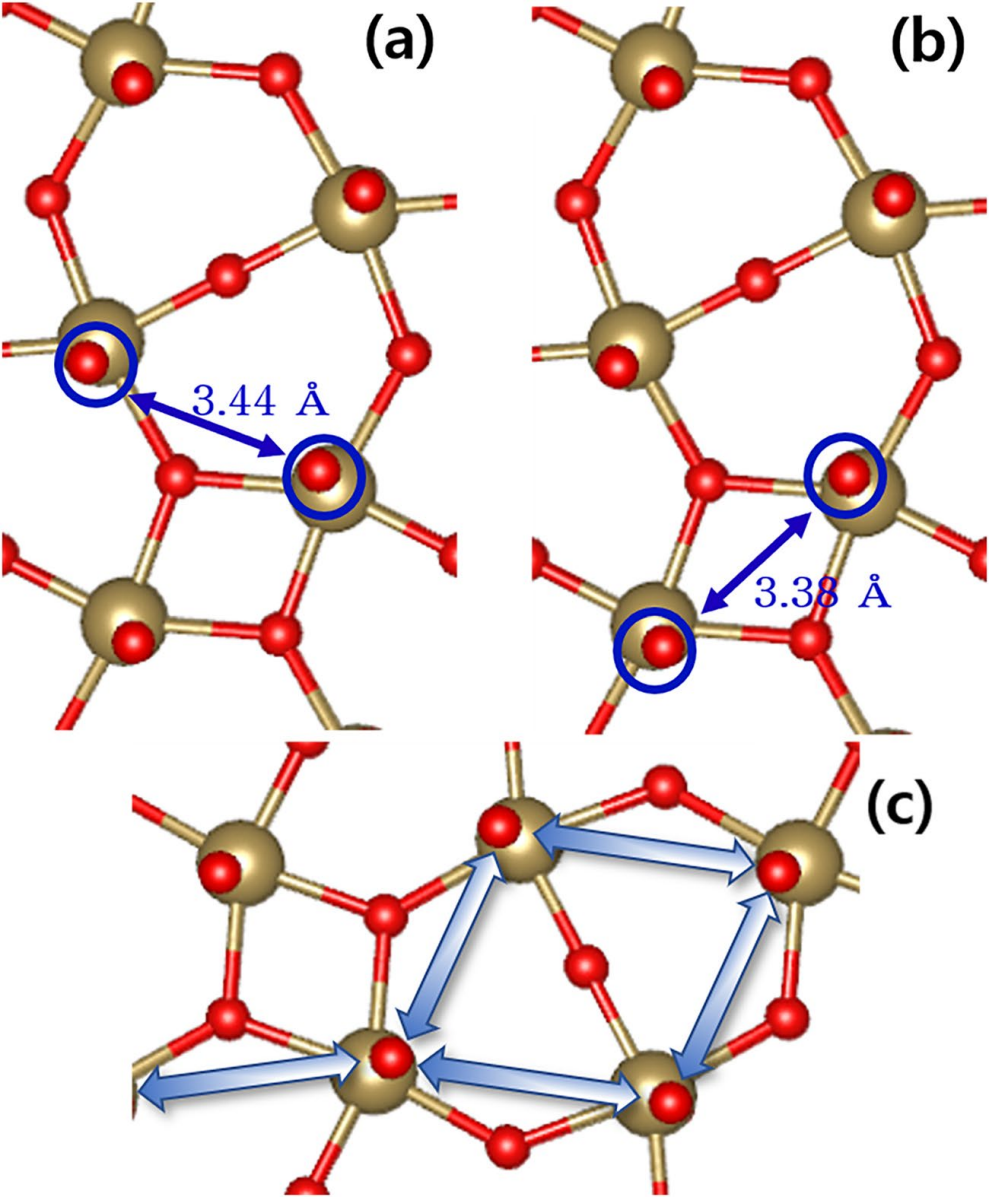
The possible O pairs participating vacancy migrations between the inter-layer O sites. Between (**a**) the second nearest inter-layer (A-path), (**b**) the first nearest inter-layer (B-path) sites. (**c**) represents one example the self-connected pathway of A-path.

**Figure 4:**
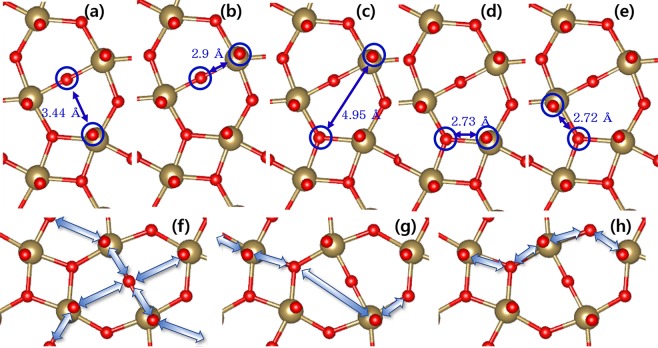
The possible O pairs participating vacancy migrations between the inter- and intra-layer O sites. (**a**) (A-path) & (**b**) (B-path) are between 2-fold intra-layer and inter-layer, (**c**–**e**) (A~E-paths) are between 3-fold intra- and inter-layer O sites. (**f**–**h**) give examples of the self-connected pathways combining (**f**) A- and B-paths, (**g**) C- and D-paths, and (**h**) D- and E-paths.

From Figs. 2–4, we show all the cases for O vacancy migrations between the nearest neighbor O atom sites drawn with the perfect cell between the intra-layer O vacancy sites (Fig. 2), the inter-layer O vacancy sites (Fig. 3), and the intra-layer and inter-layer O vacancy sites (Fig. 4). The straight-line distances between the site pairs are also marked in the figures. For O vacancy migration to have a practical meaning for applicable devices, O vacancy must be able to diffuse from one end to the other of the cell to make a voluminal change of O vacancy density in a real oxide film. Therefore, we focus our attention on pathways that can stretch in a particular direction which we call ‘self-connected path’. One can figure out there are two kinds of such pathways for between intra-layer O vacancies (A- and B-path in Fig. 2) and only one for between the inter-layer O vacancy migrations (A-path in Fig. 3). All the inter-layer to intra-layer migration pathways become self-connected by the combination of two of them (A-path + B-path, C-path + D-path, D-path + E-path in Fig. 4).

### Activation energy of O vacancy for various pathways

In ref.^12^, the calculated total energy variations during the electrically neutral O vacancy migrations along the minimum energy path (MEP) were given obtained by the nudged elastic band (NEB) method for all the possible pathways^12^. The barrier heights of the representative paths for each migration categories are 1.28 eV, 2.17 eV, and 1.65 eV for between intra-layer sites, between inter-layer sites, and between intra- and inter-layer sites respectively. Here is a point to remark that for migration between 2-fold intra- and inter-layer sites (A-path in Fig. 4), due to the energy asymmetry of the initial and final states, there is a directional preference. That is if O vacancy moves from the 2-fold intra-layer site to the neighboring 2-fold inter-layer site, the barrier is about 1.93 eV, on the other hand, for the opposite direction it reduces to about 1.65 eV which means O vacancy can move much easily from the 2-fold inter-layer to the 2-fold intra-layer than in the opposite direction. This directional preference is a phenomenon that occurs only in between inter- and intra-layer migrations and not shown in other pathway categories. One conclusion from the results is that since the barrier between inter-layer sites is much larger than the other pathways, all the plausible O vacancy migrations can occur only within Ta_2_O_3_ layer or between inter- and intra-layer sites. Of course, there is a chance that O vacancy migrations occur through grain boundaries, whose characteristics of those are hard to predict, and the smaller grain sizes are, the greater the likelihood is. However, in a later discussion about actual resistance changing volume in Ta_2_O_5_-based OxRAM, we will explain that this possibility is safely excluded.

### O vacancy formation energy for different charge states

The results described above only deal with the electrically neutral O vacancies. However, it is well known that many types of defects may prefer particular charge state depending on the formation energies of the defects and electron’s Fermi energy. In addition, formation energy is also affected by relative richness of each atomic species, as expressed by corresponding chemical potentials. Because our interest is a relative easiness of formation of O vacancies for different charge states, so here, we look at the relative formation energies of O vacancies that eliminates dependence on chemical potential of each species. Then, the relative formation energies of O vacancy states can be determined from the following expression^20^

1 $${{\rm{E}}}_{f}({V}_{O}^{q})={\rm{E}}({V}_{O}^{q})-{\rm{E}}({V}_{O}^{0})+q({{\rm{E}}}_{V}+{{\rm{\mu }}}_{e})$$

Where ${\rm{E}}({V}_{O}^{q})$ is the total energy of O vacancy with charge q, ${\rm{E}}({V}_{O}^{0})$ is the total energy of the neutral inter-layer O vacancy, E_V_ is the valence band maximum energy, and μ_e_ is the Fermi energy of electrons. Here, any additional term delivered to correct interactions between charged defects are excluded because it has become clear that the frequently employed Makov-Payne correction^21^ often significantly overestimates and less accurate than the uncorrected results^22^.

**Figure 5:**
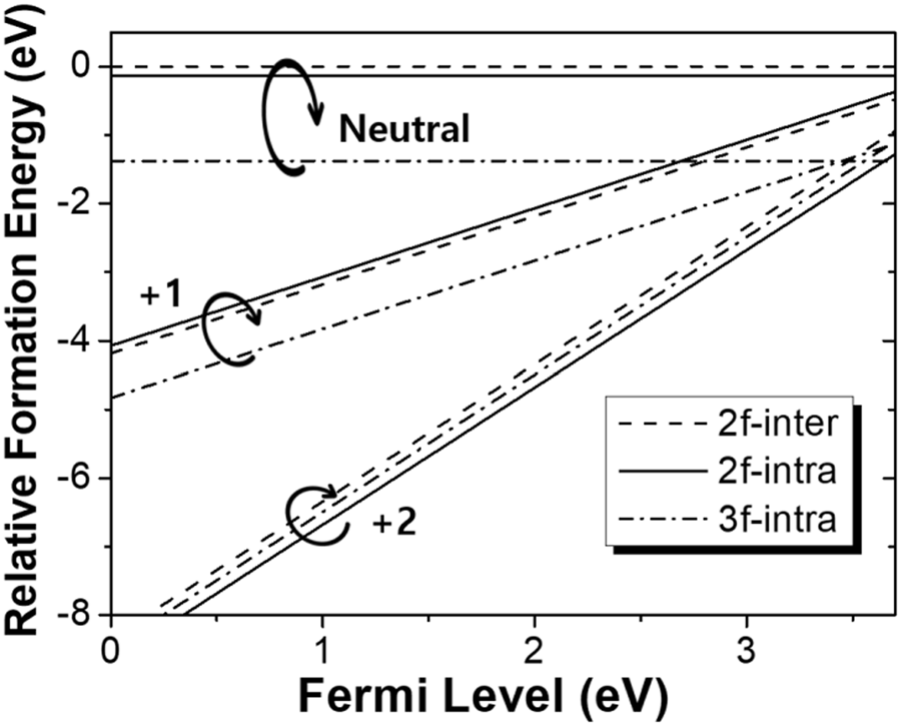
The relative O vacancy formation energy of three O sites for neutral, +1, and +2 charged states for the band gap range of 3.7 eV^18^.

We show in Fig. 5 the calculated relative formation energies of three kinds of O sites for different charge states compared to that of the neutral 2-fold inter-layer O vacancy. The range of electron Fermi energy was selected to lie in the bandgap of the orthorhombic λ phase Ta_2_O_5_. From the results, it is clear that for all the O vacancy configurations the +2 charged states can be formed much easier than the neutral or +1 charged states for almost all the Fermi energies in the band gap. That means it is certain that O vacancies are most likely to be +2 charged regardless of their configuration types. Then, the study for the activation barrier must be extended and focused to the case of +2 charged O vacancies.

### Charge state dependent activation energy of O vacancy

Generally, a fabricated Ta_2_O_5_ film having size of much larger than several hundreds of nanometers is most likely a polycrystalline or an amorphous that means lots of single crystalline grain of stacked Ta_2_O_3_ 2D layers are mixed in random angles as observed by the *in situ* transmission electric microscope (*in situ* TEM)^23^. In this situation, there is a possibility that O vacancies migrate through grain boundaries which is much likely if the corresponding activation barrier is smaller than the other pathways and degree of crystallization is low enough so that grain boundaries occupy large enough volume. In fact, the extent to which the grain boundary plays a role in O vacancy migration can vary considerably, depending on the oxide film fabrication processes. The measured O vacancy activation barriers reported so far are quite different ranging from 0.74 to 1.63 eV^11,13,14^ which are, interestingly, inversely proportional to the fabrication temperature of the Ta_2_O_5_ films. This is maybe due to, because grain size in a polycrystalline film is inversely proportional to the annealing temperature^24^, increased grain size reduces probability of O vacancy migrating through grain boundaries.

**Figure 6:**
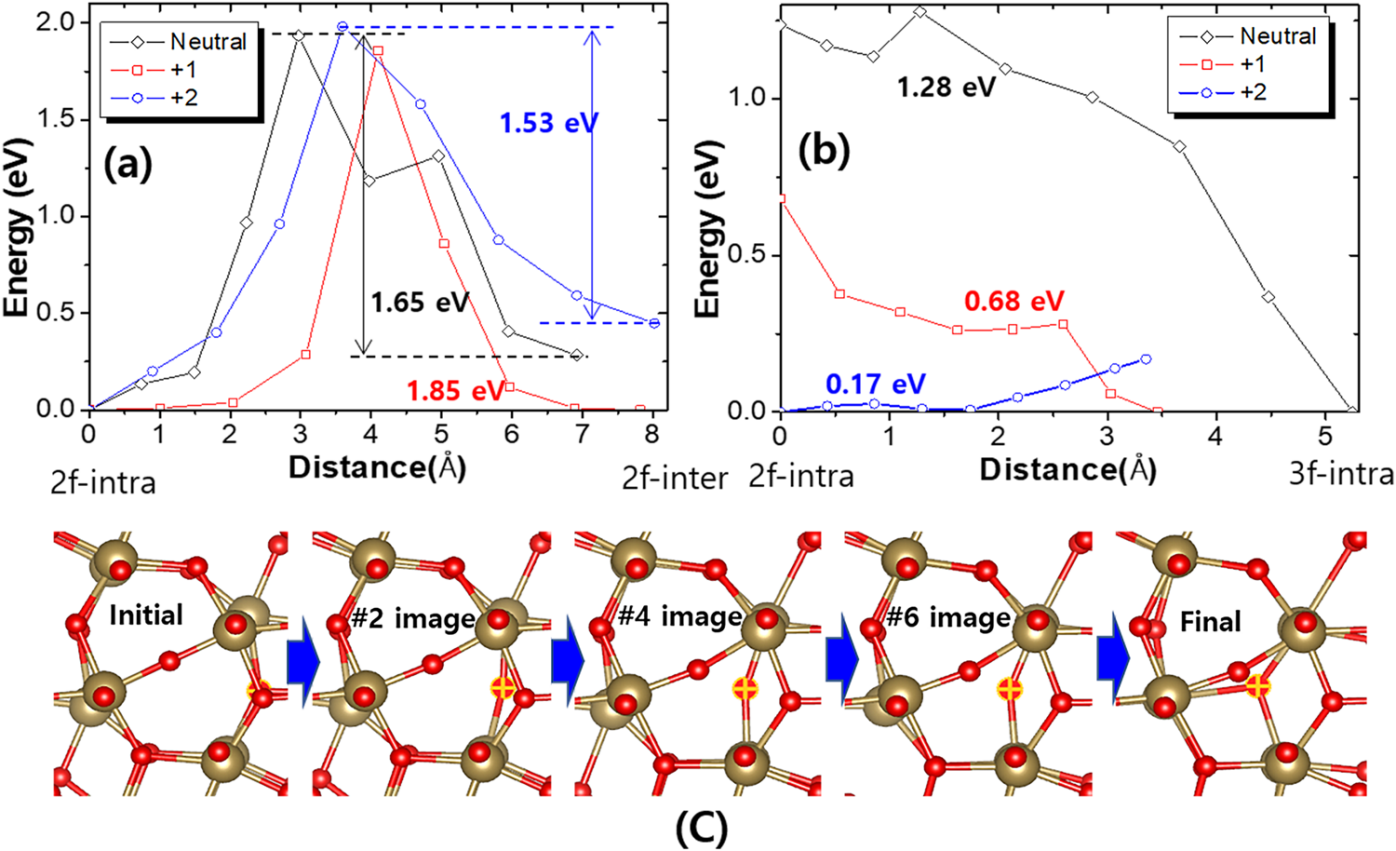
The relative total energy variation during (**a**) the O vacancy migration between the 2-fold intra-layer and the inter-layer sites (A-path in Fig. 4, 0 distance is the 2-fold intra-layer site and the right ends correspond to the inter-layer site) and (**b**) the intra-layer migration (A-path in Fig. 2, 0 distance is the 2-fold intra-layer site and the right ends correspond to the 3-fold site) for the neutral, +1, and +2 charged O vacancies. (**c**) The calculated evolution of atomic positions for the +2 charged case shown in (**b**) where the initial, final, and three out of seven images are given.

If the degree of crystallization of a Ta_2_O_5_ film is high enough, volume ratio occupied by grain boundaries becomes small, and the overall O vacancy migration is described by migrations through multiple grains, i.e. inter-layer migrations of which representative pathway is A-path (Fig. 4(a)). In Fig. 6(a), we show the total energy variation for the MEP of A-path of between the inter- and intra-layer O sites migration with the different charge states of O vacancy. The activation barrier changes slightly with charge state and for the +2 case, which we are interested in, is about 0.12 eV lower (1.53 eV) than the neutral. It is interesting this value closely matches the measured value (1.63 ± 0.17 eV) by the ^18^O tracer diffusion method^14^.

However, for a less than a few tens of nanometer oxide film like resistance changing region in an OxRAM, the situation can be quite different. For Ta_2_O_5_-based OxRAM, because a few nanometer thickness, resistance switching Ta_2_O_5−x_ layer is deposited on a near amorphous TaO_2−x_ layer, it inevitably has various crystal orientations depending on the location^3–5,8^. In this Ta_2_O_5−x_ layer thus deposited, prior to resistance switching operations, a filament that actual resistance changes occur is formed by causing an electrical breakdown at somewhere in the film by applying a large electric field. As with all electrical breakdown phenomena, a breakdown occurs in the spot within the plane of the Ta_2_O_5−x_ film where the O activation barrier is the lowest and defective for O atoms to migrate. As discussed above, O vacancies might travel through grain boundaries that might have a lower activation barrier. However, the filament of Ta_2_O_5_-based OxRAM has been revealed to be voluminal having a cross-sectional area of about 30 nm^2^ ^8^. Furthermore, it is impossible to account for the high on-current of the OxRAM (up to 100 mA) solely by the phase change (even if it is possible) within grain boundaries of which width is one or two lattice constant at most. Therefore, even if O vacancy migration through grain boundary occurs during filament forming process, voluminal collective migration of O vacancies within single crystal grain(s) that defines the actual filament region and represents the whole migration characteristic must be accompanied.

In this situation, therefore, it is most likely that a filament of a Ta_2_O_5_-based OxRam is formed at the spot where Ta_2_O_3_ layers are aligned in the electric field direction so that O vacancy migrations can occur most easily. And then, as explained above, O vacancies have the strongest tendency to go through along the pathway between the 2-fold and the 3-fold intra-layer sites that has the lowest O activation barrier of all.

Now, we should look into how the activation barrier on this pathway is affected by the charge state of O vacancy especially for +2. Figure 6(b) shows the relative total energy variation during O vacancy migrations with different charge states for MEP of this pathway. It is remarkable that, unlike the case of inter-layer migration shown in Fig. 6(a), the activation barrier is monotonically and significantly lowered from 1.28 eV to 0.68 eV and finally to 0.17 eV as the charge state changes sequentially from 0, +1, and +2. No experimental measurement has been made in this nanometer regime. However, the activation barrier in Ta_2_O_5_ film to this level of tininess can be indirectly deduced from the operating characteristic of the Ta_2_O_5_-based OxRAM. From the theoretical model of OxRAM operation that describes O vacancy migration considering the barrier lowering by electric field within lattice, avalanche type migration of O vacancies occur when the electric field grows to the extent of image force barrier lowering term, 2E_A_/q_O_d^5,6^, where E_A_ is the O vacancy activation barrier, q_O_ is the charge state of O vacancy (+2), and d is the straight-line distance between the 2-fold and the 3-fold intra-layer sites which is about 0.27 nm. Combining this model and the results from the experimental reports of Ta_2_O_5_-based OxRAMs showing the abrupt resets (from low to high resistance state switching) occur at around 2 V with about 3 nm Ta_2_O_5_ film thicknesses^2–5^, the activation energy (E_A_) is estimated to be ~0.18 eV which is quite close to the calculation (0.17 eV). The low activation barrier in this highly reproducible Ta_2_O_5_-based OxRAMs suggests again that it is likely to be due to the intrinsic properties of Ta_2_O_5_ crystal, not by random factors, which, according to our study, can only be explained by the intra-layer migrations of +2 charged O vacancies.

The characteristics that appear in this intra-layer O vacancy migration can be understood as follows: Unlike the case of inter-layer migration shown in Fig. 6(a), the electrical potential saddle structure on the pathway that forms bell-shaped barrier does not turn up for the pathway between the 2-fold and 3-fold intra-layer sites, instead, a nearly monotonic change in total energy throughout the path appears. Since the valence band maximum state is formed by p-orbital of inter-layer and 3-fold intra-layer O atoms^18^, the total energy is mainly determined by the p-orbital overlapping interaction between these. For the inter-layer migration pathway, the migrating O atom initially located at the inter-layer site with the initial distance of 2.73 Å comes close to the 3-fold intra-layer O atom within 2.27 Å at the midpoint along the (17% closer). Consequently, this close approach and orbital overlap makes the total energy higher and results in the bell-shaped barriers as shown in Fig. 6(a). On the other hand, for migration pathway between 2-fold and 3-fold intra-layer sites, two O atoms come to close only within 2.68 Å from the initial distance of 2.74 Å (2% closer) and makes little change in total energy. As a result, monotonical potential variations along the pathway appear and barrier is determined simply by the initial and final configurations which goes down to 0.17 eV for +2 charged O vacancy. In this occasion, bell-shaped potential barriers emerge when considering migration pathways involving an additional step, i.e. 2-fold intra → 3-fold → 2-fold intra or 3-fold → 2-fold intra → 3-fold and the exceptionally low activation barrier for +2 O vacancy is due to the coincidental similarity in energy of the two configurations.

**Figure 7:**
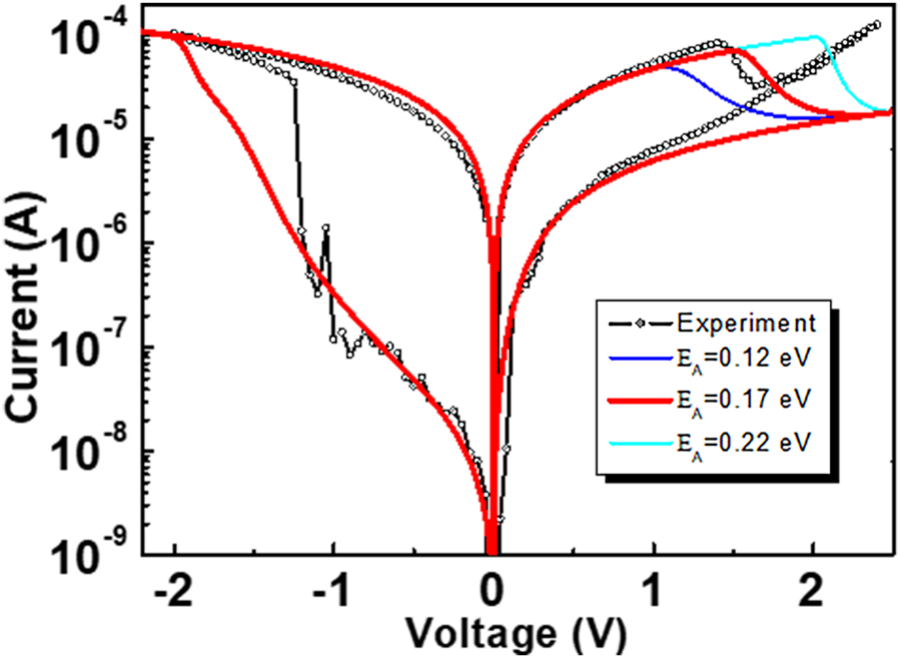
Comparisons between the I-V curves from the OxRAM switching model with different values of E_A_ (0.12 eV, 0.17 eV, and 0.22 eV) and the experimental I-V curve. The model parameters are the same with those described in ref.^4^.

Finally, it would be useful to reconfirm how well the calculated activation barrier fits with the experimental result when applied to the OxRAM switching model^5^. From the model, the mobility of O vacancy (μ_OV_) has the following relation with activation barrier (E_A_):

2 $${{\rm{\mu }}}_{{\rm{OV}}}\propto \exp \,[-({{\rm{E}}}_{{\rm{A}}}-\frac{{{\rm{q}}}_{{\rm{o}}}{\rm{Ed}}}{2})/{\rm{kT}}],$$

where q_O_ is the charge state of O vacancy (=2), E is the electric field, and d is the O vacancy migration distance. Inserting Eq. (2) into the OxRAM switching model described in ref.^5^, we can obtain model I-V curves as a function of the specific structural and material parameters. By comparing the model results with different activation barriers, we can see the calculated E_A_ is the representative of very low E_A_ characteristic expected by the OxRAM switching model. In Fig. 7, we show the comparative plot of I-V curves with different values of E_A_ and the typical experimental I-V curve of the Ta_2_O_5_ based OxRAM obtained from the same conditions described in ref. ^4^. From this figure, it is clear the calculated O vacancy activation barrier lies within the best-suited area for the experimental result.

### Summary

A comprehensive study on activation energy of O vacancy in the orthorhombic λ phase Ta_2_O_5_ via first-principles DFT calculations has been made. The study was aimed to understand the origin of the exceptionally small O vacancy activation barrier estimated from the operational characteristics of Ta_2_O_5_-based OxRAMs. We calculated activation barriers for almost all possible scenarios and found pathways from which to determine the overall O vacancy migration characteristics. It was found that, in the case of Ta_2_O_5_-based OxRAMs, a resistance changing volume is most likely to be formed at a location where Ta_2_O_3_ layers are aligned with the filament forming electric field. In this case, +2 charged O vacancies can move much freely with the very small activation barrier of 0.17 eV. This low activation barrier of O vacancy originated from the inherent nature of the orthorhombic λ phase Ta_2_O_5_, rather than by uncontrollable elements such as grain boundaries or dislocations, allows Ta_2_O_5_-based OxRAM to operate at low voltages and low power consumption with excellent reproducibility.

## Methods

The first-principles calculations are based on Kohn-Sham theory and the projector-augmented wave potentials as implemented in the Quantum ESPRESSO package^20^. For Ta the 6 s and 5d orbitals were treated as valence states and the Perdew, Burke, and Ernzerhof (PBE) potential with a core radius of 2.84 a.u was used and for O, the PBE potential with a core radius of 1.55 a.u. was applied. Valence electron wavefunctions were expanded in a planewave basis set with a cutoff energy of 400 eV. We use, for all the calculations, the 2 × 2 × 2 supercell expanded from the unit cell of the orthorhombic λ phase Ta_2_O_5_^17–19^ which comprises with 111 atoms for 1 O vacancy. The ion relaxation calculations with an O vacancy that used for the initial and final structure of migration barrier calculations, were performed with the k-points generated with a mesh spacing 0.25 Å^−1^ applying generalized gradient approximated (GGA) exchange-correlation functional under Hellmann-Feynman force criterion of 0.02 eV/Å.

The migration pathway of the O vacancy and the corelated energy barrier were determined by finding the minimum energy path (MEP) from one lattice site to an adjacent site using a variation of the nudged elastic band (NEB)^25–27^ method as implemented in Quantum ESPRESSO. First, the two endpoint (initial and final) configurations of interested vacancy states were determined by separately optimized ion relaxation calculations. At these two states, it was assumed that the energies are in local minima and the forces applied at atoms are nearly zero. Since the MEP lies between two end states, firstly, a set of intermediate atomic configurations so called images are generated with constant spacing between the two endpoints, and this whole forms an ‘elastic band’. Then, finding the MEP is carried out, while keeping the two endpoints fixed, by nudging the elastic band little by little toward zero forces. In other words, for the images to reach the MEP, each image must be moved toward MEP by a vector of forces at each step. This forces mainly consist of interatomic forces due to displacement of atoms and an artificial spring force that added by connecting two adjacent images with a spring with a constant spring constant. This virtual spring force works only in a direction parallel to the line that connects the images and makes the images pass through the energy minimum that passes through the saddle point, not the real energy minimum and interatomic forces only act in the direction perpendicular to the band. More specifically, we used NEB climb method^25^ designed for one of images to be positioned at the saddle point of potential. This method minimizes the possibility of the NEB process reaching an incorrect path other than MEP. We adopted 7 images for all the calculations by means of a parallel calculation implementation of the Quantum ESPRESSO code, with each image assigned to a separate processor core.

## Data Availability

The data that support the findings of this study are available from the corresponding author upon reasonable request.
